# m6A-immune-related lncRNA prognostic signature for predicting immune landscape and prognosis of bladder cancer

**DOI:** 10.1186/s12967-022-03711-1

**Published:** 2022-10-29

**Authors:** Zi-Hao Feng, Yan-Ping Liang, Jun-Jie Cen, Hao-Hua Yao, Hai-Shan Lin, Jia-Ying Li, Hui Liang, Zhu Wang, Qiong Deng, Jia-Zheng Cao, Yong Huang, Jin-Huan Wei, Jun-Hang Luo, Wei Chen, Zhen-Hua Chen

**Affiliations:** 1 Department of Urology, The First Affiliated Hospital, Sun Yat-sen University, Guangzhou, Guangdong China; 2 Department of Urology, Affiliated Longhua People’s Hospital, Southern Medical University, Shenzhen, Guangdong China; 3 Department of Urology, Jiangmen Central Hospital, Jiangmen, Guangdong China

**Keywords:** m6A (N6-methyladenosine), Long noncoding RNA (lncRNA), Bladder cancer (BLCA), Immune microenvironment, Immune cell infiltration, Prognosis prediction

## Abstract

**Background:**

N6-methyladenosine (m6A) related long noncoding RNAs (lncRNAs) may have prognostic value in bladder cancer for their key role in tumorigenesis and innate immunity.

**Methods:**

Bladder cancer transcriptome data and the corresponding clinical data were acquired from the Cancer Genome Atlas (TCGA) database. The m6A-immune-related lncRNAs were identified using univariate Cox regression analysis and Pearson correlation analysis. A risk model was established using least absolute shrinkage and selection operator (LASSO) Cox regression analyses, and analyzed using nomogram, time-dependent receiver operating characteristics (ROC) and Kaplan–Meier survival analysis. The differences in infiltration scores, clinical features, and sensitivity to Talazoparib of various immune cells between low- and high-risk groups were investigated.

**Results:**

Totally 618 m6A-immune-related lncRNAs and 490 immune-related lncRNAs were identified from TCGA, and 47 lncRNAs of their intersection demonstrated prognostic values. A risk model with 11 lncRNAs was established by Lasso Cox regression, and can predict the prognosis of bladder cancer patients as demonstrated by time-dependent ROC and Kaplan–Meier analysis. Significant correlations were determined between risk score and tumor malignancy or immune cell infiltration. Meanwhile, significant differences were observed in tumor mutation burden and stemness-score between the low-risk group and high-risk group. Moreover, high-risk group patients were more responsive to Talazoparib.

**Conclusions:**

An m6A-immune-related lncRNA risk model was established in this study, which can be applied to predict prognosis, immune landscape and chemotherapeutic response in bladder cancer.

**Supplementary Information:**

The online version contains supplementary material available at 10.1186/s12967-022-03711-1.

## Introduction

Bladder cancer is the 10th most common malignancy worldwide, resulting in about 170,000 deaths worldwide every year [1]. Though numerous studies have been conducted, and great improvement has been made in last decades, the 5-years survival rate remains unsatisfactory. Therefore, identification of novel biomarkers for diagnosis and personalized treatment bladder cancer is of great importance.

Long noncoding RNAs (lncRNAs) with a length of more than 200 nucleotides, generally could not translate into proteins. lncRNAs participate in cell growth, differentiation and proliferation by regulating gene expression both transcriptionally and post-transcriptionally [2–4].

As the most common RNA modification, N6-Methyladenosine (m6A) occurs not only in messenger RNAs (mRNAs), but also in lncRNAs [5, 6]. m6A methylation affects nearly all the RNA metabolism aspects, including RNA translocation, splicing, stabilization, and translation [7, 8]. The m6A modification is mediated by three types of m6A regulators, including m6A-binding proteins (readers), demethylases (erasers), and methyltransferases (writers) [9].

Previous reports showed that dysregulated expression of lncRNAs is critical in tumorigenicity and metastasis of bladder cancer [10, 11]. For instance, lncRNA BLACAT2 was reported to be able to promote bladder cancer lymphatic metastasis, and blocking VEGF-C signaling with a VEGF-C antibody reduced LN metastasis of high BLACAT2- expressing bladder cancers in vivo [12]. LncRNA PTENP1 was reported to suppress bladder cancer progression [13]. Lnc-LBCS was found inhibit self-renewal, chemoresistance, and tumor initiation of bladder cancer stem cells through epigenetic silencing of SOX2 both in vitro and in vivo [14]. m6A-induced lncDBET was reported to promote the malignant progression of bladder cancer through FABP5-mediated lipid metabolism in vitro and in vivo [15]. Moreover, increasing research has used lncRNAs as biomarker in predicting response in bladder cancer treatment, including ferroptosis-related, m6A-related, and immune-related lncRNA models [16–18]. However, most of these studies did not validate their models by conducting cell biology experiments.

In this study, the m6A-immune-related lncRNAs with prognostic value for construction of a risk model were identified, and the correlations between the risk model and the immune microenvironment were investigated.

## Materials and methods

### Data source and retrieve

The mutation data, public transcriptome data, and the corresponding bladder cancer clinical information were obtained from TCGA (https://portal.gdc.cancer.gov/) database, and CIBERSORT immune fractions of these data were obtained from https://gdc.cancer.gov/about-data/publications/panimmune. The RNA sequence transcriptome data and GSE154261 clinical data were obtained from GEO. m6A regulators, including readers (LRPPRC, RBMX, HNRNPA2B1, HNRNPC, FMR1, YTHDF3, YTHDF1, YTHDF2, IGF2BP2, IGF2BP3, IGF2BP1, YTHDC1, and YTHDC2), writers (ZC3H13, METTL3, RBM15, RBM15B, VIRMA, WTAP, METTL16, and METTL14), erasers (FTO and ALKBH5), and were obtained from published articles.

### Identification of m6A-immune-related lncRNAs

Totally 618 m6A-immune-related lncRNAs were identified by Pearson’s correlation analysis between lncRNAs and m6A regulators. Correlations between lncRNAs and CIBERSORT immune fractions were analyzed and 490 immune-related lncRNAs were identified. Through Univariate Cox proportional hazard regression analysis, 49 lncRNAs were extracted, and the intersections of these lncRNAs were used for further analysis.

### Construction and validation of an m6A-immune-related lncRNA risk model

LASSO Cox regression was used for analysis of 47 shared prognostic m6A-immune-related lncRNAs, and 11 of them were ultimately used for construction of a risk model. The risk score was determined on the basis of the multiplication of lncRNA expression and each coefficient. The genomic location and correlation of these lncRNAs and m6A regulators were visualized by “circlize” package in R software. Patients were classified into low-risk group and high-risk group. The differences between high- and low-risk groups were analyzed in multiple dimensions, including clinical features, expression levels of these lncRNAs and m6A regulators, immune infiltration estimations (TIMER [19], CIBERSORT [20] and TCIA [21]), cell stemness index [22], tumor mutation burden and commonly mutated genes. Visualization was conducted by “pheatmap”, “ggplot2” and “maftools” package.

### Validation of m6A-immune-related lncRNA risk model

The prognostic capability of the risk model was evaluated using the Kaplan–Meiler survival curve. The specificity and sensitivity of the risk model were evaluated using the area under curve (AUC). The validation was performed on GSE154261 dataset.

### Gene set enrichment analysis (GSEA) and pathway annotation

GSEA was performed in R software by using “clusterprofiler” package, and revealed the associated signaling pathways. Visualization of pathways was done by using “pathview” and “ggnet” packages.

### Cell lines and cell culture

T24 and RT-112, two bladder cancer cell lines, were purchased from ATCC and cultured in RPMI-1640 medium supplemented with 10% FBS.

### Wound healing and invasion assays

Cells were seeded into six-well plate and scratched with a pipette. The photos were taken at different time points after scratching. In invasion assay, Cells (1 × 10^5^) were seeded into a Boyden chamber (8 mm pore size) pre-coated with matrigel in serum-free medium, and RPMI-1640 containing 10% FBS was added to the bottom chamber. After 48 h, the filter lower surface cells were fixed, stained, and counted under a microscope.

### Colony formation assay

The IC50 of Talazoparib was calculated based on dose–response growth curve. 200 untreated cells were seeded into each well of six-well plate, and cultured with or without Talazoparib for 2 weeks, and the colonies were then analyzed.

### Statistical analysis

All data processing and statistical analysis was performed in R software (version 4.1.0). P value < 0.05 was considered statistically significant.

## Results

### Identification of m6A-immune-related lncRNAs in bladder cancer

**Fig. 1:**
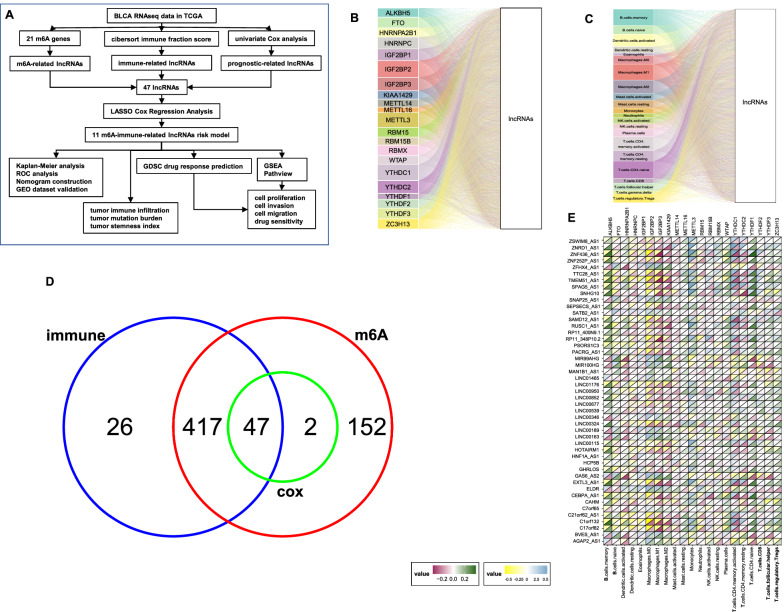
Flow-chart and identification of m6A-immune-related lncRNAs. **A** The workflow of the current study. **B** Sankey relational diagram for 21 m6A genes and m6A-related lncRNAs. **C** Sankey relational diagram for 22 immune fractions and immune-related lncRNAs. **D** Venn diagram of selected Immune-related-, m6A-related- and prognostic-related-lncRNAs. The 47 overlapped lncRNAs were identified for further analysis. **E** The upper part of each grid shows the correlations between the 47 lncRNAs and m6A regulators, and the bottom part shows the correlations between the 47 lncRNAs and immune fractions

Figure 1A shows the workflow process. Totally 814 lncRNAs were included in this study. 618 lncRNAs were identified to be significantly correlated with at least one of the m6A regulators as analyzed by Pearson correlation analysis (Fig. 1B). Similarly, 490 lncRNAs were identified as immune-related lncRNAs as analyzed by Pearson correlation analysis between lncRNAs and CIBERSORT immune fractions (Fig. 1C). The overall survival- and progression-free survival-related prognostic was filtered using lncRNAs Univariate Cox regression analysis, and 49 lncRNAs showed prognostic values (Fig. 1D). Figure 1E shows the correlations between these 47 shared lncRNAs and CIBERSORT immune fractions as well as m6A regulators.

### Construction of m6A-immune-related lncRNA risk score and its correlation with clinicopathological characteristics

**Fig. 2:**
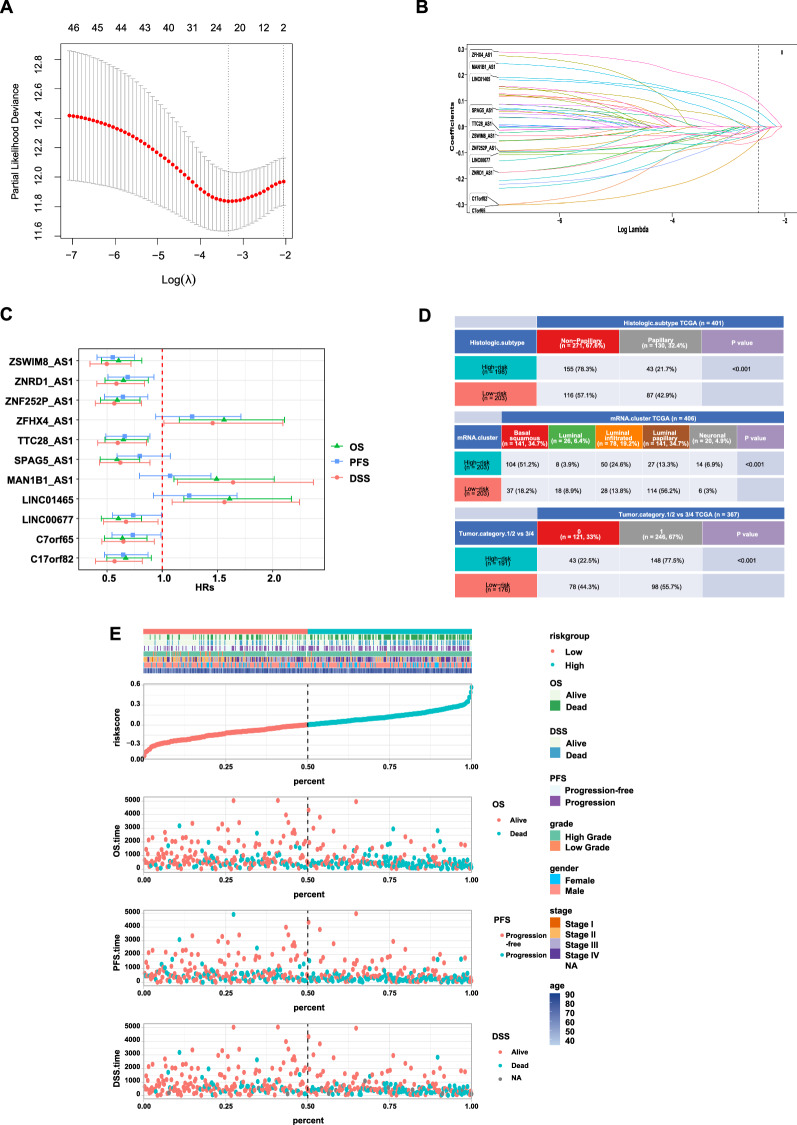
Construction of m6A-immune-related lncRNA risk score and its correlation with clinicopathological characteristics. **A** The LASSO Cox regression analysis was carried out to construct a risk model. The best parameter (λ) was selected based on the LASSO model. **B** According to the best λ, 11 lncRNAs were included and the coefficients of them are shown. **C** Forest plot of univariate Cox regression analysis of 11 m6A-immune-related lncRNAs. The Hazard Ratio (HR) value and its 95% confidence interval are shown. **D** Heatmap and table showing the distribution of risk score of patients in histology subtypes, Lund molecular classifier subtypes, and stage subgroups. **E** Distribution of clinicopathological features of m6A-immune-related lncRNAs model-based risk score

LASSO-Cox regression analysis was carried out on the shared lncRNAs that were significantly correlated to both m6A-regulators and CIBERSORT immune fractions to construct a risk score for prediction of overall survival. Figure 2A shows the risk model constructed using 11 selected lncRNAs, Fig. 2B shows the coefficient of each lncRNA, and Fig. 2C shows the forest plot of progression-free survival, overall survival, and disease-specific survival. Additional file 1: Fig. S1 shows the Kaplan–Meier plots of each lncRNA. Additional file 1: Fig. S2 shows the genomic location and correlation between these lncRNAs and m6A regulators. The risk scores were calculated, and based on the median value, they were classified to low- or high-risk subgroup. Figure 2D shows the correlations between risk score and clinicopathological characteristics. Higher risk score indicates later stage of bladder cancer patients, and most high-risk group tumors were basal squamous subtype, while most low-risk group tumors were luminal papillary subtype. Figure 2E shows the high-risk group with poorer prognosis.

### Nomogram construction and GEO dataset validation

**Fig. 3:**
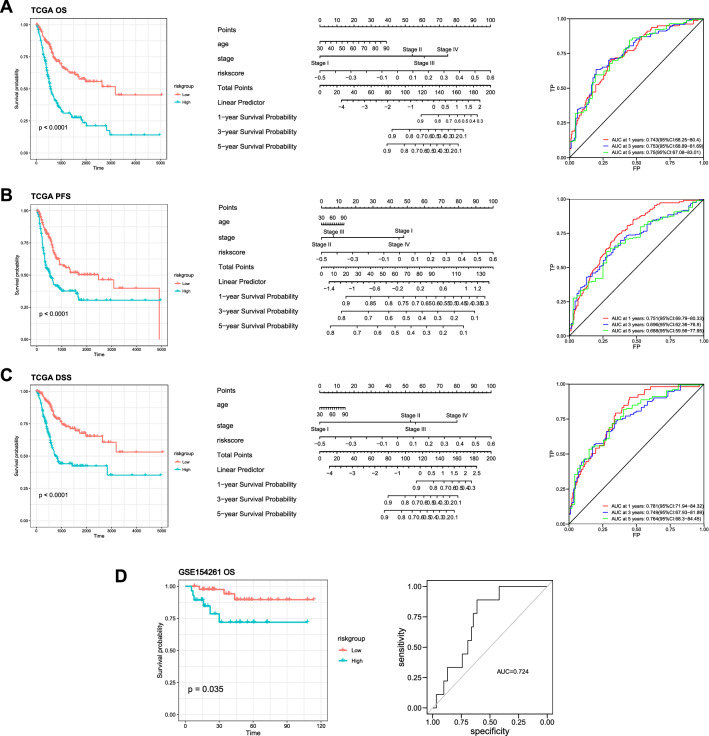
Nomogram construction and GEO dataset validation. **A**–**C** The left panel shows Kaplan–Meier plots for comparison of low-risk group and high-risk group of overall survival (**A**), progression-free survival (**B**) and disease-specific survival (**C**). The middle panel shows nomograms for comparison of the risk score, and clinical risk characteristics were fabricated to predict the 1-, 3-, and 5-year OS, PFS, and DSS incidences. 1‐, 3‐, and 5‐year ROC curves of the risk score were charted in the right panel to predict OS, PFS, and DSS. **D** The risk model was validated in independent dataset from GEO, which shows significant predictive value in OS

The reliability and sensitivity of the risk model was assessed using ROC curves. As shown in Additional file 1: Fig. S3, the risk model only dependent on risk score alone may not be sufficient for predicting patients’ prognosis. Therefore, risk score was combined with other key clinical characteristics, including age of diagnosis, pathologic tumor stage, and three comprehensive nomograms to make prediction in OS, PFS and DSS. Figure 3A–C show the Kaplan–Meier plots comparing high-risk group and low-risk group of progression-free survival, overall survival, and disease-specific survival. By using time dependent ROC analysis in “timeROC” package, the AUC of these three nomograms at 1, 3 and 5 years are shown in Fig. 3A–C. All AUCs at 1 year are larger than 0.74, indicating this model shows important prognostic value. To validate the predictive accuracy of this model, it was applied to an independent dataset, which provides overall survival data, from GEO (GSE154261). ROC analysis manifested that the AUC of this model was close to that of the TCGA dataset, suggesting this model is robust in other datasets (Fig. 3D).

### Comparison of tumor mutation rate, mutation burden and tumor immune microenvironment score between high-risk group and low-risk group

**Fig. 4:**
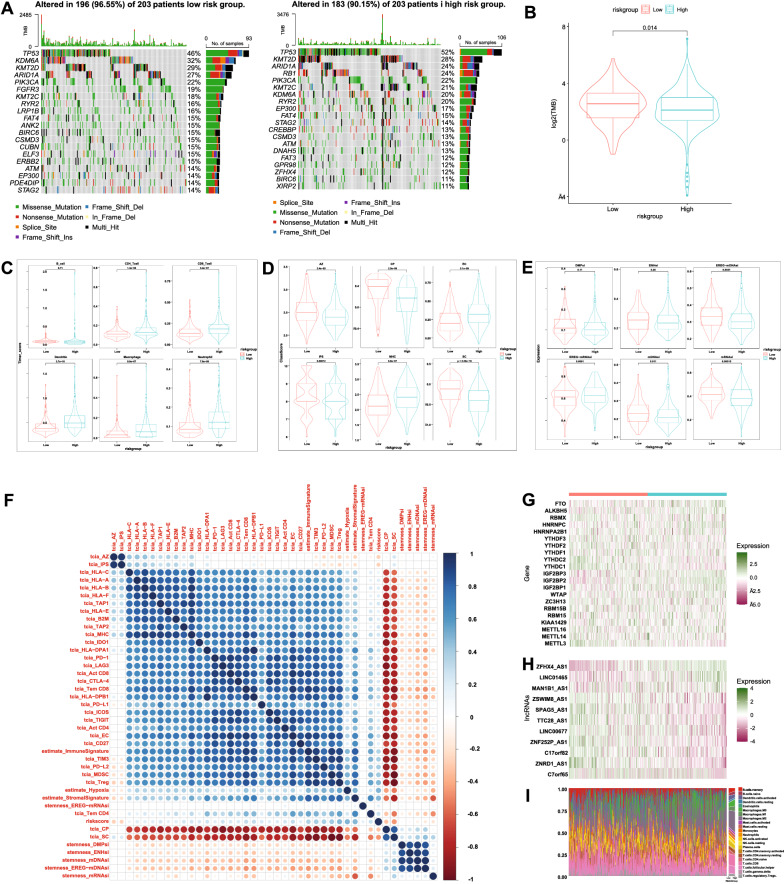
Comparisons between high-risk group and low-risk group. **A** The oncoplots of two risk subgroups showing significant mutation rate difference in some commonly mutated genes in bladder cancer. **B** Tumor mutation burden in the low-risk group was higher than that in the high-risk group(p < 0.05). **C** Higher Immunophenoscore in low-risk group indicates that the patients in low-risk group might have stronger immunogenicity and might be more sensitive to immunotherapy. **D** All types of infiltrating immune cells in high-risk group were more abundant than those in the low-risk group as calculated using the TIMER algorithm. **E** Low-risk group showed higher stemness indices than high-risk group. **F** Correlation matrix showing the relationship between risk score and immune factors. **G**–**I** 21 m6A regulator expression pattern (**G**), 11 m6A-immune-related lncRNAs expression pattern (**H**), and CIBERSORT immune infiltration pattern (**I**)

The relationships between the risk score and tumor mutation rate, mutation burden, tumor immune microenvironment score and tumor stemness indices were determined. Some commonly mutated genes in bladder cancer showed significant difference between low-risk group and high-risk group. The mutation rate of KDM6A in the low-risk subgroup was 12% lower than that in the high-risk subgroup (Fig. 4A, Additional file 1: Fig. S4). The patients in low-risk group had a higher mutation burden (Fig. 4B). Immunophenoscore was used to predict the potential response of bladder cancer patients to immune checkpoint inhibitors (ICI). The statistical analysis results showed that the immunophenoscore was larger in the low-risk group than in the high-risk group, which means patients in low-risk group might have stronger immunogenicity and might be more sensitive to immunotherapy (Fig. 4C, Additional file 1: Fig. S5). All of the infiltrating immune cells including CD4 + and CD8 + T cells, dendritic cells, neutrophils, B cells, and macrophages in the high-risk group were more abundant than those in the low-risk group as calculated using the Tumor Immune Estimation Resource (TIMER) algorithm (Fig. 4D). Interestingly, low-risk group showed higher stemness indices, suggesting cell stemness and immunogenicity play different roles in bladder cancer (Fig. 4E). Correlation matrix shows the relationship between risk score and these factors (Fig. 4F). The expression heatmap of 21 m6A regulators and 11 m6A-immune-related lncRNAs shows their relationships with risk score (Fig. 4G, H). Sorting patients by risk score, significant transition in tumor microenvironment can be observed in the heatmap of CIBERSORT fractions (Fig. 4I). The infiltration percentage of macrophage in the low-risk group was remarkably lower than that in the high-risk group, while naïve T cells shrank in high-risk group.

### GSEA and pathway correlation analysis

**Fig. 5:**
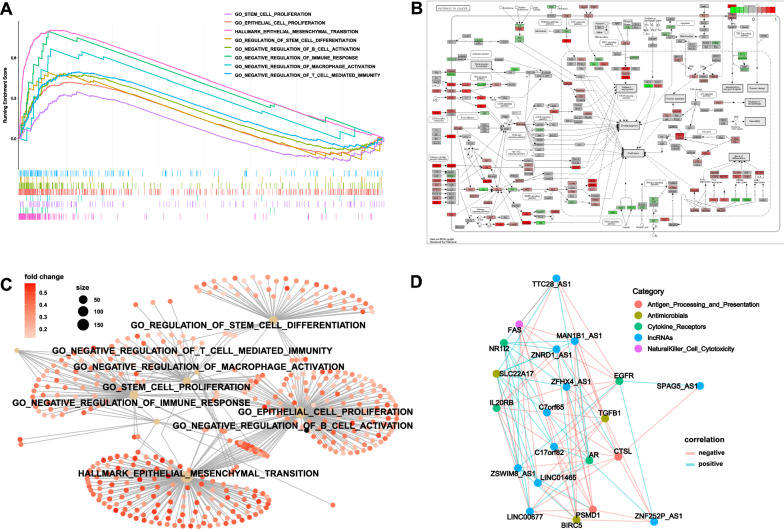
GSEA and pathway correlation analysis. **A** Gene set enrichment analysis showing that these lncRNAs were related to immune process regulation, cell proliferation and EMT. **B** Pathview analysis showing that these lncRNAs may take part in multiple pathways in cancer development and progression. **C** Correlation network of pathways showing that they were tightly connected. **D** The coexpression network revealing the relationship between m6A-immune-related lncRNAs and immune regulating genes in bladder cancer

GSEA was conducted to identify the pathways associated with this risk model. As shown in Fig. 5A,“GO_STEM_CELL_PROLIFERATION”,“GO_EPITHELIAL_CELL_PROLIFERATION”,“HALLMARK_EPITHELIAL_MESENCHYMAL_TRANSITION”,“GO_REGULATION_OF_STEM_CELL_DIFFERENTIATION”,“GO_NEGATIVE_REGULATION_OF_B_CELL_ACTIVATION”,“GO_NEGATIVE_REGULATION_OF_IMMUNE_RESPONSE”,“GO_NEGATIVE_REGULATION_OF_MACROPHAGE_ACTIVATION”, and “GO_NEGATIVE_REGULATION_OF_T_CELL_MEDIATED_IMMUNITY” were significantly positively correlated to the risk score. This result suggests that the model was highly related to tumor proliferation, migration, invasion and tumor immune microenvironment. The relationships between these pathways were visualized by using “Pathview” and “ggnetwork” package in R (Fig. 5B, C). The relationships between 11 lncRNAs and their most correlated immune genes were also visualized (Fig. 5D).

### Drug sensitivity analysis in GDSC and in vitro validation

**Fig. 6:**
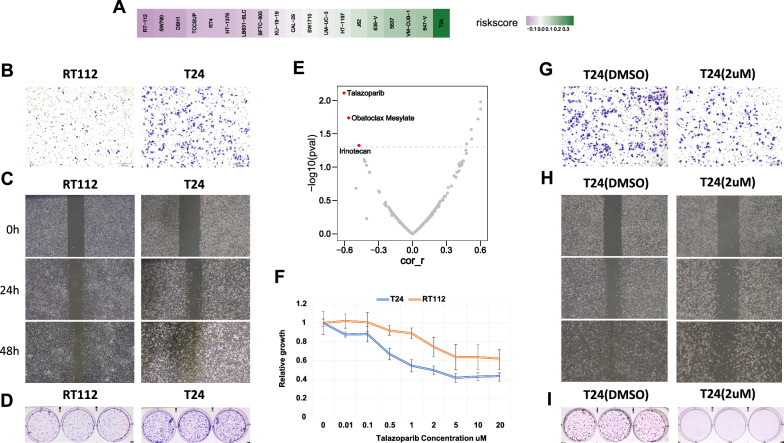
Drug sensitivity analysis and in vitro validation. **A** Application of risk model to CCLE bladder cancer cell lines sorted by their risk score. **B**–**D** Invasion assay (**B**), wound healing assay (**C**) and colony formation assay (**D**) showing that T24 had relatively higher malignancy than RT112. **E** Volcano plot showing that cell lines with higher risk score were more sensitive to Talazoparib.** F** Dose-response growth curve of Talazoparib in T24 and RT112. **G**–**I** Validation of sensitivity to Talazoparib in vitro showing that T24 cell line with a higher risk score, was more sensitive to Talazoparib

The risk model was applied to CCLE, and the risk score of bladder cancer cell line was calculated. As shown in Fig. 6A, T24 had the highest risk score while RT-112 had the lowest risk score), consistent with previous studies of bladder cancer, which indicated that T24 had relatively higher malignancy than other cell lines [23–25]. We further validated these results in vitro by performing colony formation, wound healing, and invasion assays (Fig. 6B–D). Furthermore, by applying drug sensitivity data in GDSC, we performed Pearson correlation analysis between cell line risk scores and the drug IC50 values. A significantly negative correlation was identified between risk score and IC50 of Talazoparib, which means patients with higher risk score are more sensitive to Talazoparib (Fig. 6E). The IC50 (2 µM) of Talazoparib in T24 was calculated (Fig. 6F), and colony formation assay was performed, which showed that T24 cells were sensitive to Talazoparib (Fig. 6G–I). This might provide a theoretical basis for targeted therapy of bladder cancer.

## Discussion

Accumulating studies have focused on the lncRNAs and m6A modifications in tumorigenesis, tumor progression and innate immunity [26]. m6A modification is the most frequent and plentiful RNA modification form, which plays critical roles in cancer development via regulation of m6A demethylases, methyltransferases, and binding proteins [27]. A close correlation between m6A regulators and lncRNAs was found, and cellular biological functions and expression of target genes can be regulated by interaction between lncRNAs and m6A regulators [6]. However, the correlation between m6A modification of lncRNAs and bladder cancer remains unclear. In this study, 11 m6A-immune-related lncRNAs were identified based on the TCGA dataset, and a risk model was constructed that was closely correlated with clinicopathological features, including molecular subtype, tumor mutation burden, tumor stage, and tumor immune microenvironment. The model was further validated in independent dataset and a series of in vitro experiments were performed. The results of this study might help understand the m6A modifications in cancer progression as well as the antitumor immune response, which might highlight the potential of this model in target therapy and immunotherapy of bladder cancer.

The m6A modification is critical in the pathological processes of cancer development [28], and lncRNAs can regulate the expression of m6A regulators [29]. For instance, lncRNA THAP7-AS1, which exerts oncogenic functions in gastric cancer, was transcriptionally activated by SP1 and then stabilized by METTL3-mediated m6A modification [30]. In esophageal squamous cell carcinoma, up-regulation of LINC00022 mediated by FTO promotes cell proliferation and tumor growth [31]. ZNF252P-AS1, which is involved in our risk model, has been demonstrated to facilitate ovarian cancer progression via miR-324-3p/LY6K signaling [32]. ZNRD1-AS1, another m6A-immune-related lncRNA involved in our prognostic model, has been found to enhance both gastric cancer cell proliferation and metastasis of nasopharyngeal carcinoma [33].

In recent years, transcriptome profiling based molecular subtyping of bladder cancer, including muscle-invasive and non-muscle-invasive, has shown promise for predicting outcomes and response to therapy and several molecular classifications have been proposed [34, 35]. The TCGA mRNA molecular classifier is the most frequently used classifier to determine the treatment response and prognosis of muscle-invasive bladder cancer though there are debate and challenge on its clinical application [36]. In our study, high risk score was correlated with basal squamous subtype, while low risk score was correlated with luminal papillary subtype, consistent to prior report that basal squamous subtype shows poorer prognosis than other molecular subtypes [36]. Additionally, low-risk group showed a higher tumor mutation burden, which is in agreement with previous studies that genomic unstable bladder cancers are more responsive to immune checkpoint inhibitor treatment [37]. These findings suggest that our model has prognostic significance and can provide insights into the crosstalk between m6A modification and molecular characteristics in bladder cancer.

As GSEA analyses revealed, enriched pathways and hallmarks were mostly tumorigenesis-related, metastasis-related, and immune-related, such as cell proliferation, EMT, macrophage activation, and immune response. m6A modification has been demonstrated to play a key role in tumor progression and immune microenvironment. The tumor immune microenvironment, which has received extensive attention recently, is always in dynamic change and its imbalance can lead to the generation and development of several types of cancer [38, 39]. In our study, the expression of m6A patterns was significantly associated with CIBERSORT immune fractions and the ESTIMATE score. Enhanced infiltration of M2 macrophages, which has been accepted as a key contributor to progression of tumor and poor outcomes [40–42], was found in the high-risk score group. The naïve CD4 + T cells and memory B cells in the high-risk score group decreased dramatically when compared with those in the low-risk group. These results indicate that m6A modification patterns are highly associated with TME cell infiltration, which may provide insights for individualized therapies by determining the response to immunotherapy.

Our risk model was validated not only in independent dataset from GEO, but also in cell lines from CCLE. According to the expression data obtained from CCLE, the risk scores calculated by our model were consistent with the malignancies of cell lines. By performing the Pearson correlation analysis of the risk score of cell lines and drug sensitivities, it was found that risk score was markedly positively correlated with Talazoparib sensitivity, suggesting that m6A-immune-related lncRNAs might be valuable in guiding more effective target therapies.

Undeniably, this study has some limitations. The first one is the insufficiency in mechanism elucidation of these m6A-immune-related lncRNAs. How they function in shaping the TME and promoting tumor growth and progression remains unclear and needs further study. Moreover, a larger sample size is needed to further validate this model. Like most of the transcriptome based molecular classification, this study is based on tumor sample after patients undergo invasive procedure. Body fluids such as saliva in cancer diagnostics and classification should receive due attention [43]. Despite these limitations, our study has identified m6A-immune-related lncRNAs and successfully established a risk model for predicting survival and response to immunotherapy and target therapy.

In conclusion, from the TCGA-bladder cancer cohort, 11 m6A-immune-related lncRNAs were identified and a risk score model was constructed with robust prognostic value. This model can predict response to immunotherapy in bladder cancer patients. Our findings provide a critical insight into the functions of m6A-immune-related lncRNAs in bladder cancer tumorigenesis, progression, and tumor immune microenvironment construction.

## Supplementary Information

**Additional file 1: Figure S1.** OS, PFS, and DSS Kaplan–Meier plots of 11 lncRNAs cutting off by their median expression, respectively. **Figure S2.** Chord diagram showing the genomic location and correlation of 11 lncRNAs and 21 m6A regulators. Red links indicate positively correlated, while bule link indicate negatively correlated. **Figure S3.** ROC analysis of the risk model. Left-top panel shows the accuracy of risk score model predicting OS, PFS and DSS. The other three panels show time-dependent ROC analysis of this model in predicting OS, PFS, and DSS in 1, 3, and 5 years. **Figure S4.** Chord diagram showing the genomic location of frequently mutated genes in bladder cancer. Red and blue circles show the mutation rate of these genes in low- and high-risk groups, respectively. **Figure S5.** Comparation between low- and high-risk groups of all factors in TCIA.

## Data Availability

Any reasonable requests for access to available data underlying the results reported in this article will be considered. Such proposals should be submitted to the corresponding author.
