# Supplementary figures and images for: m6A-immune-related lncRNA prognostic signature for predicting immune landscape and prognosis of bladder cancer
Source: J Transl Med. 2022 Oct 29;20:492. doi: 10.1186/s12967-022-03711-1 (PMC9617388; doi:10.1186/s12967-022-03711-1)

Figure S1

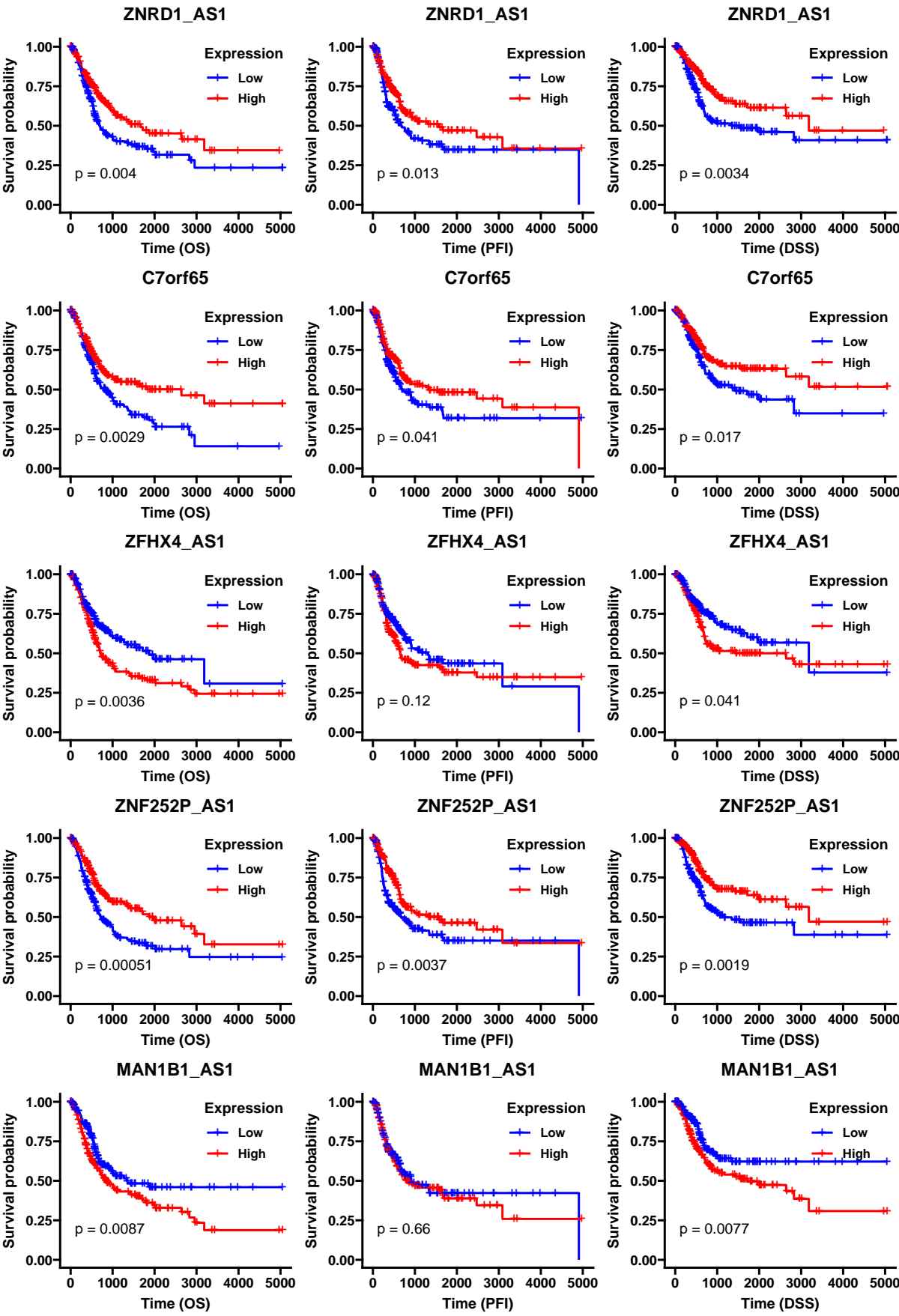

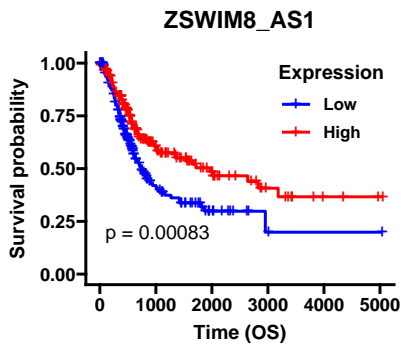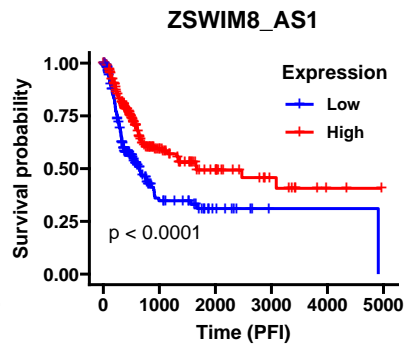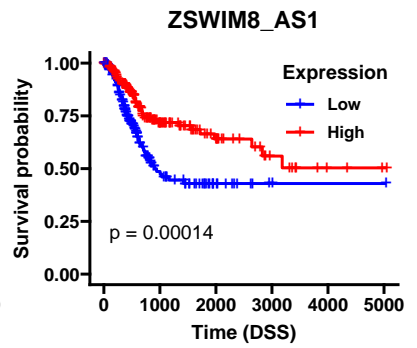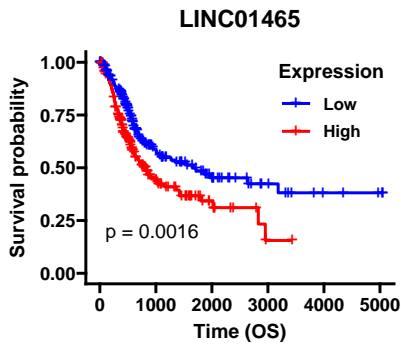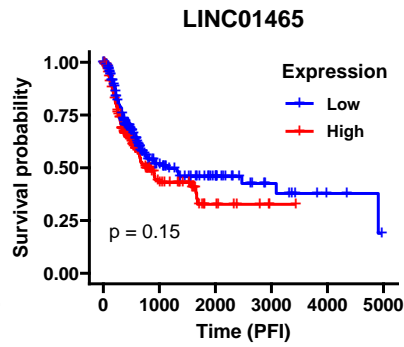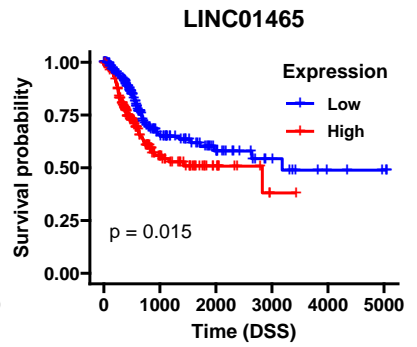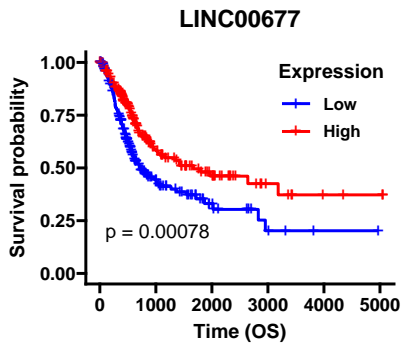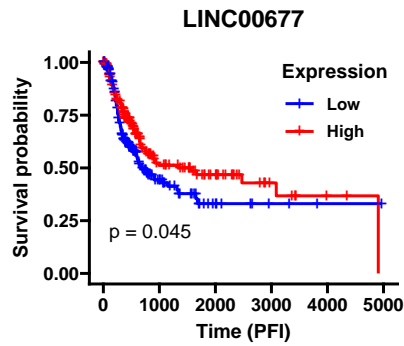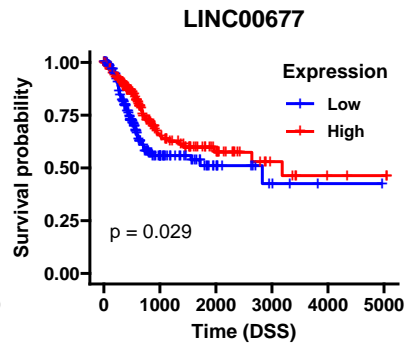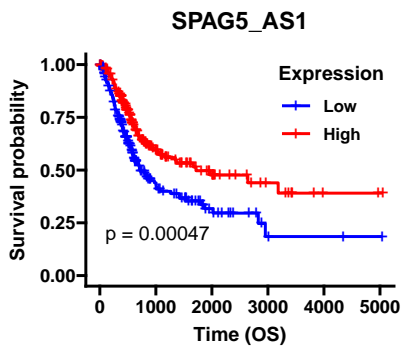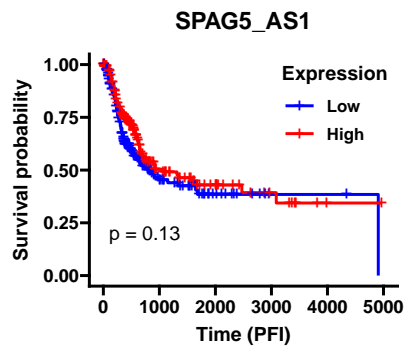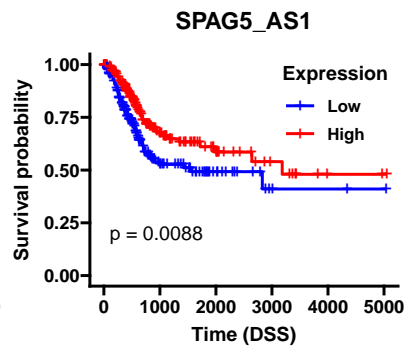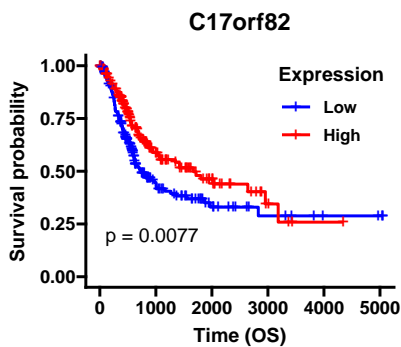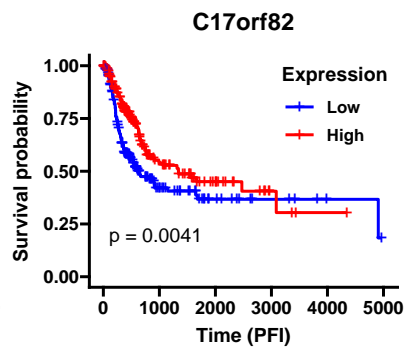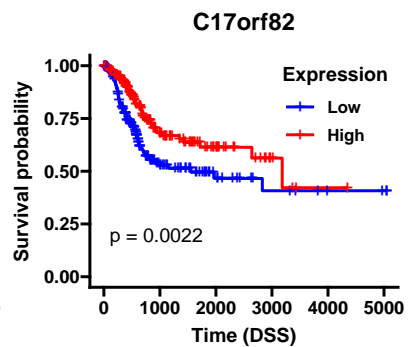

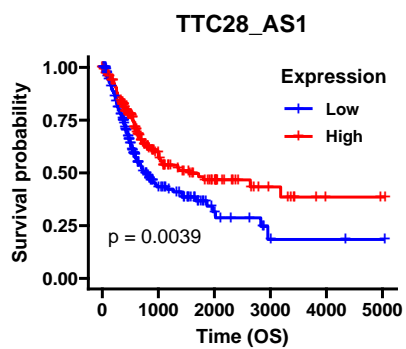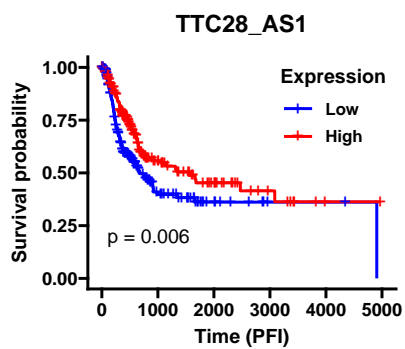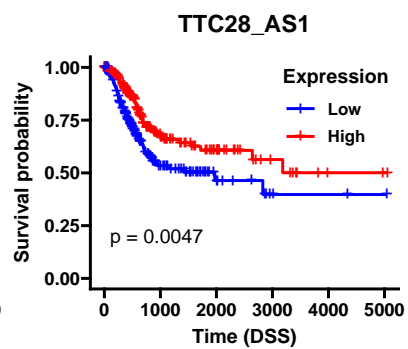

Figure S2

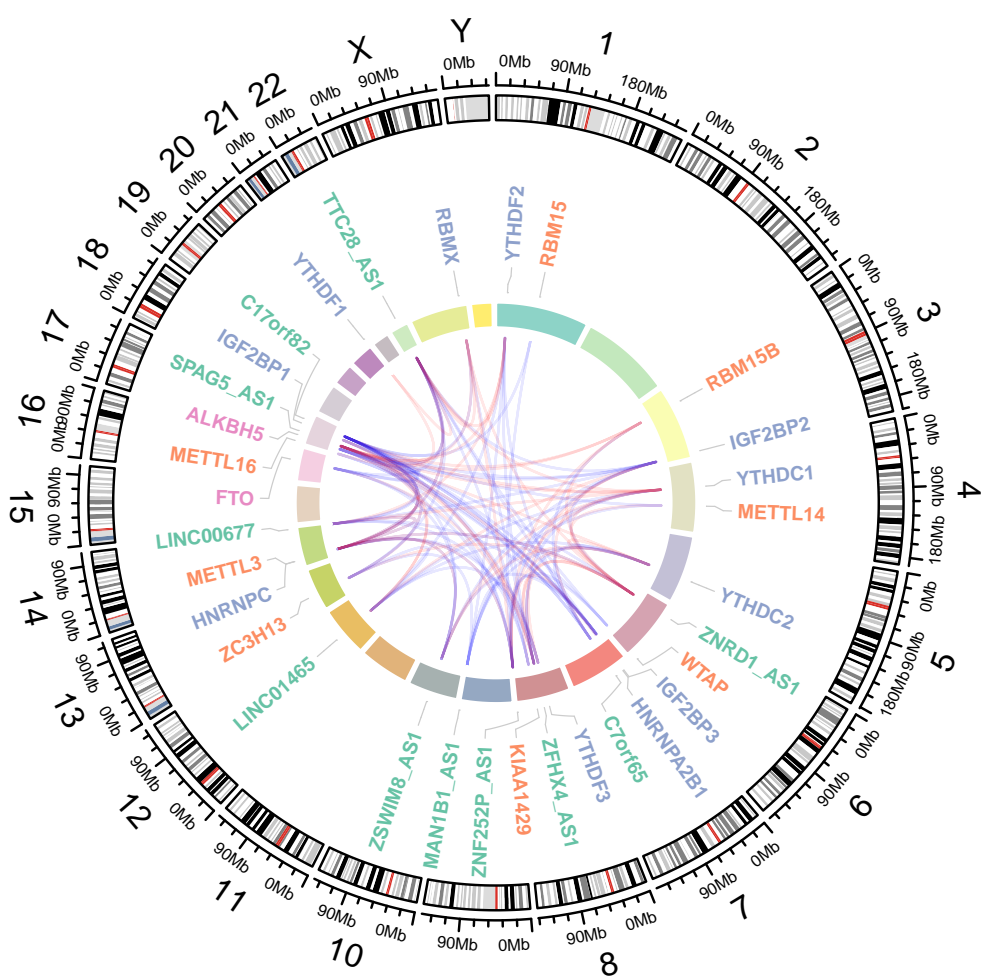

Figure S3

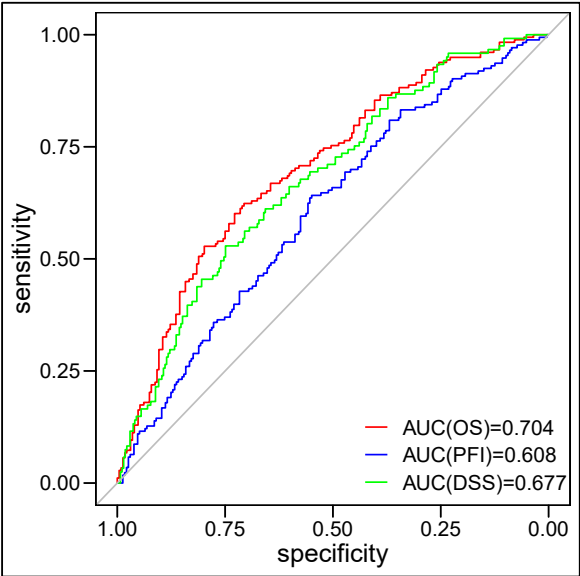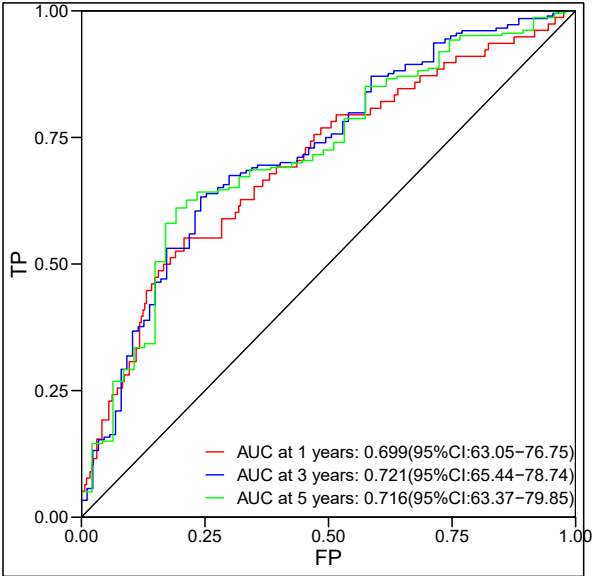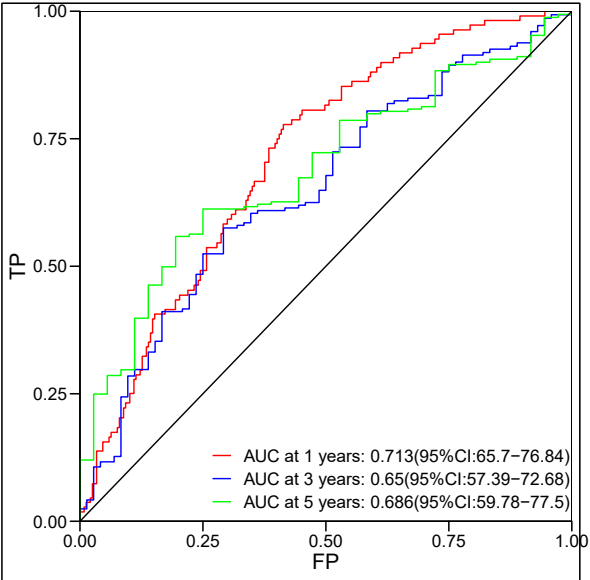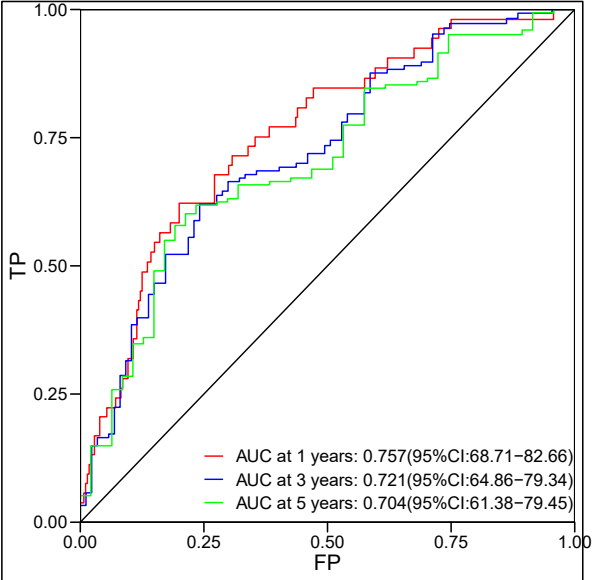

Figure S4

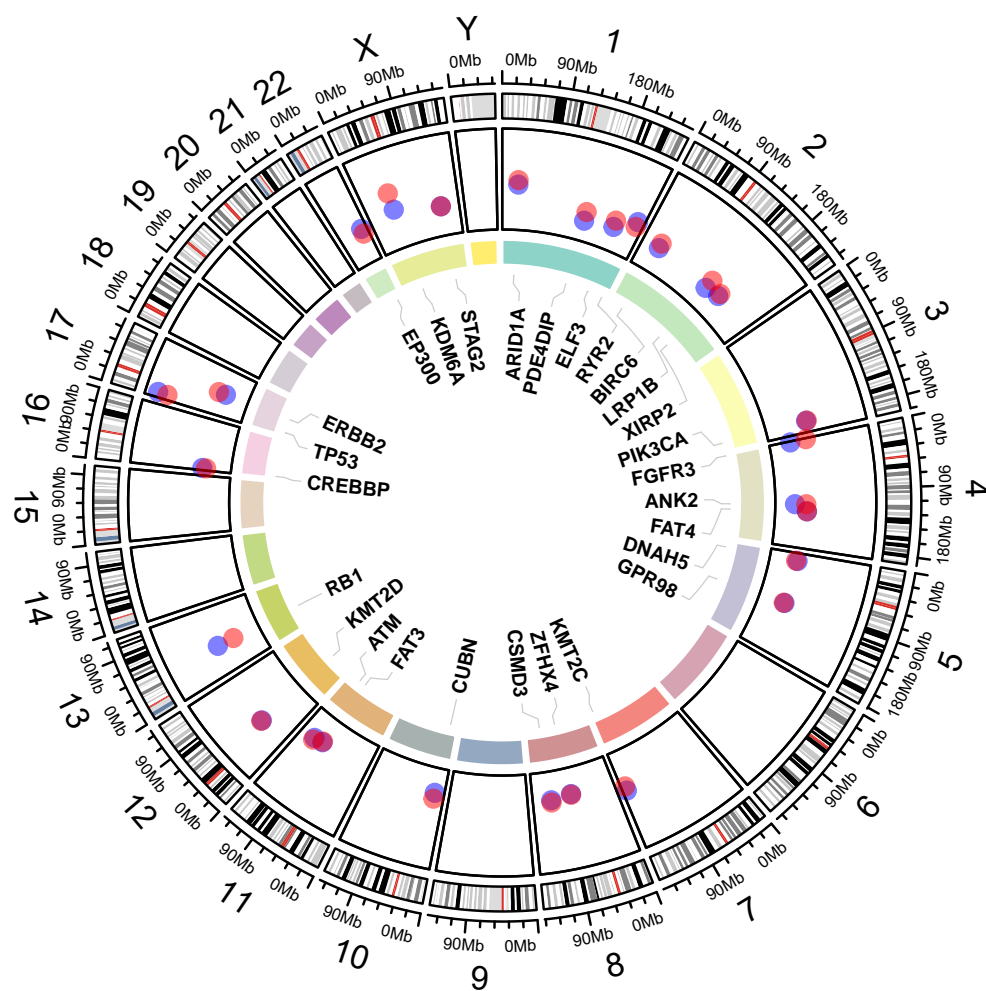

Figure S5

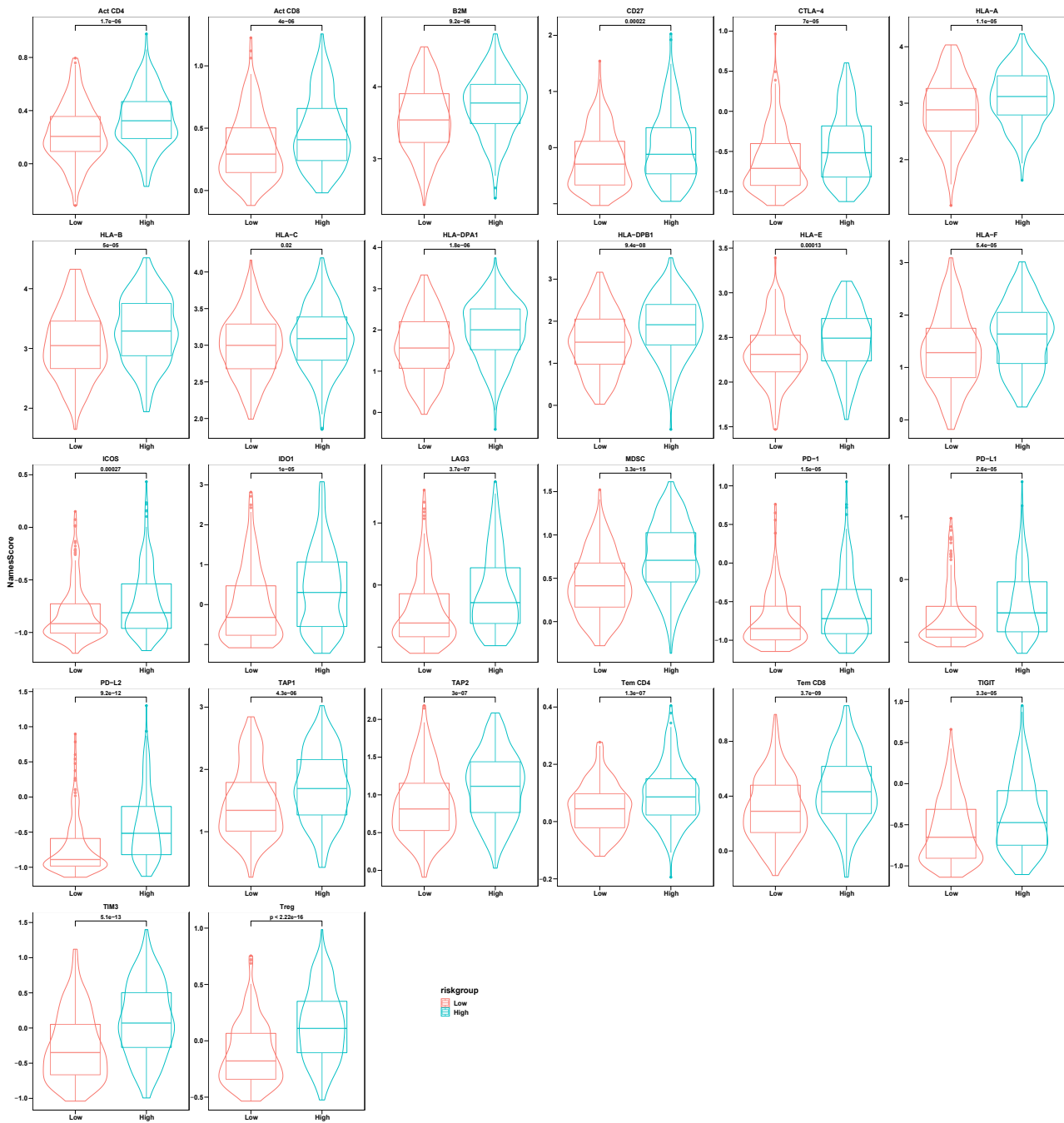

Supplement: Supplementary file 1 — Additional file 1: Figure S1. OS, PFS, and DSS Kaplan–Meier plots of 11 lncRNAs cutting off by their median expression, respectively. Figure S2. Chord diagram showing the genomic location and correlation of 11 lncRNAs and 21 m6A regulators. Red links indicate positively correlated, while bule link indicate negatively correlated. Figure S3. ROC analysis of the risk model. Left-top panel shows the accuracy of risk score model predicting OS, PFS and DSS. The other three panels show time-dependent ROC analysis of this model in predicting OS, PFS, and DSS in 1, 3, and 5 years. Figure S4. Chord diagram showing the genomic location of frequently mutated genes in bladder cancer. Red and blue circles show the mutation rate of these genes in low- and high-risk groups, respectively. Figure S5. Comparation between low- and high-risk groups of all factors in TCIA. [file 12967_2022_3711_MOESM1_ESM.pdf]
